# Evaluation of three-dimensional acromiohumeral distance in the standing position and comparison with its conventional measuring methods

**DOI:** 10.1186/s13018-020-01935-9

**Published:** 2020-09-23

**Authors:** Yuki Yoshida, Noboru Matsumura, Yoshitake Yamada, Minoru Yamada, Yoichi Yokoyama, Morio Matsumoto, Masaya Nakamura, Takeo Nagura, Masahiro Jinzaki

**Affiliations:** 1 Department of Orthopedic Surgery, Keio University School of Medicine, 35 Shinanomachi, Shinjuku-ku, Tokyo, 160-8582 Japan; 2 Department of Radiology, Keio University School of Medicine, 35 Shinanomachi, Shinjuku-ku, Tokyo, 160-8582 Japan

**Keywords:** Normal shoulder, Acromiohumeral distance, Acromiohumeral interval, Upright computed tomography, Position, Digitally reconstructed radiographs

## Abstract

**Background:**

Narrowing of the acromiohumeral distance (AHD) implies a rotator cuff tear. However, conventional AHD measurements using two-dimensional (2D) imaging or with the patient in the supine position might differ from that while standing during daily activity. This study aimed to evaluate the three-dimensional (3D) actual distance between the acromion and humeral head in the standing position and compare the AHD values with those obtained using conventional measuring methods.

**Methods:**

Computed tomography (CT) images of 166 shoulders from 83 healthy volunteers (31 male and 52 female; mean age 40.1 ± 5.8 years; age range, 30–49 years) were prospectively acquired in the supine and standing positions using conventional and upright CT scanners, respectively. The minimum distance between the acromion and humeral head on the 3D surface models was considered as the 3D AHD. We measured the 2D AHD on anteroposterior digitally reconstructed radiographs. The AHD values were compared between the supine and standing positions and between the 2D and 3D measurements.

**Results:**

The mean values of 2D AHD were 8.8 ± 1.3 mm (range, 5.9–15.4 mm) in the standing position and 8.1 ± 1.2 mm (range, 5.3–14.3 mm) in the supine position. The mean values of 3D AHD were 7.3 ± 1.4 mm (range, 4.7–14.0 mm) in the standing position and 6.6 ± 1.2 mm (range, 4.4–13.7 mm) in the supine position. The values of 3D AHD were significantly lower than those of 2D AHDs in both the standing and supine positions (*P* < 0.001). The values of 2D and 3D AHDs were significantly lower in the supine position than in the standing position (*P* < 0.001).

**Conclusions:**

This study evaluated the 3D AHD of normal shoulders in the standing position using an upright CT scanner. The present results indicated that assessments in the supine position can underestimate the value of the AHD compared with those made in the standing position and that assessments using 2D analysis can overestimate the value.

## Introduction

The acromiohumeral distance (AHD) is usually assessed on anteroposterior shoulder radiographs acquired in the standing position. Narrowing of this distance is widely considered to imply a rotator cuff tear [1–4]. However, the distances on radiographic two-dimensional (2D) measurements might differ from the actual distance between the humeral head and acromion [5]. Although a three-dimensional (3D) distance can be measured using computed tomography (CT), conventional CT scanners acquire images of the shoulder girdle only in the supine position [6, 7]. Moreover, it is possible that the AHD could change between the supine and standing positions owing to the effect of gravity and the rotation of the upper arm.

The minimum distance between the acromion and humeral head in the standing position has not been evaluated using a 3D approach. However, a novel upright CT scanner has been developed, which enables 3D whole-torso cross-sectional scanning in the standing position [8] and evaluation of the effect of gravity on the human body [9]. This study aimed to clarify the 3D minimum distance between the acromion and humeral head of normal shoulders in the standing position using an upright CT scanner and compare the AHD values with those obtained using conventional methods. We hypothesized that 3D measurement of the actual distance between the acromion and humeral head in the standing position is feasible using CT scans and that its AHD values differ from those obtained using 2D analysis or in the supine position.

## Materials and methods

### Participants

This study was approved by the institutional review board, and written consent was obtained from all the participants (study protocol: #20160384). The study was conducted between June 2017 and July 2018.

A total of 134 healthy male and female volunteers aged 30–89 years with no past illnesses or injuries to the shoulder girdle were recruited from a volunteer recruitment company. Of these, 50 participants aged over 50 years were excluded because of the increased risk of an asymptomatic rotator cuff tear or other degenerative changes in the shoulder girdle [10–12]. In addition, one volunteer aged 33 years was excluded because of defects in the shoulder observed on the CT image. Thus, 166 shoulders from 83 healthy volunteers (31 male and 52 female) were included in the analysis. The participants’ mean (± standard deviation) age, height, body weight, and body mass index (BMI) were 40.1 ± 5.8 years (range, 30–49 years), 162.9 ± 8.7 cm (range, 147.7–184.0 cm), 59.6 ± 12.5 kg (range, 37.8–106.8 kg), and 22.4 ± 3.7 kg/m^2^ (range, 15.7–33.7 kg/m^2^), respectively.

### Image acquisition

**Fig. 1:**
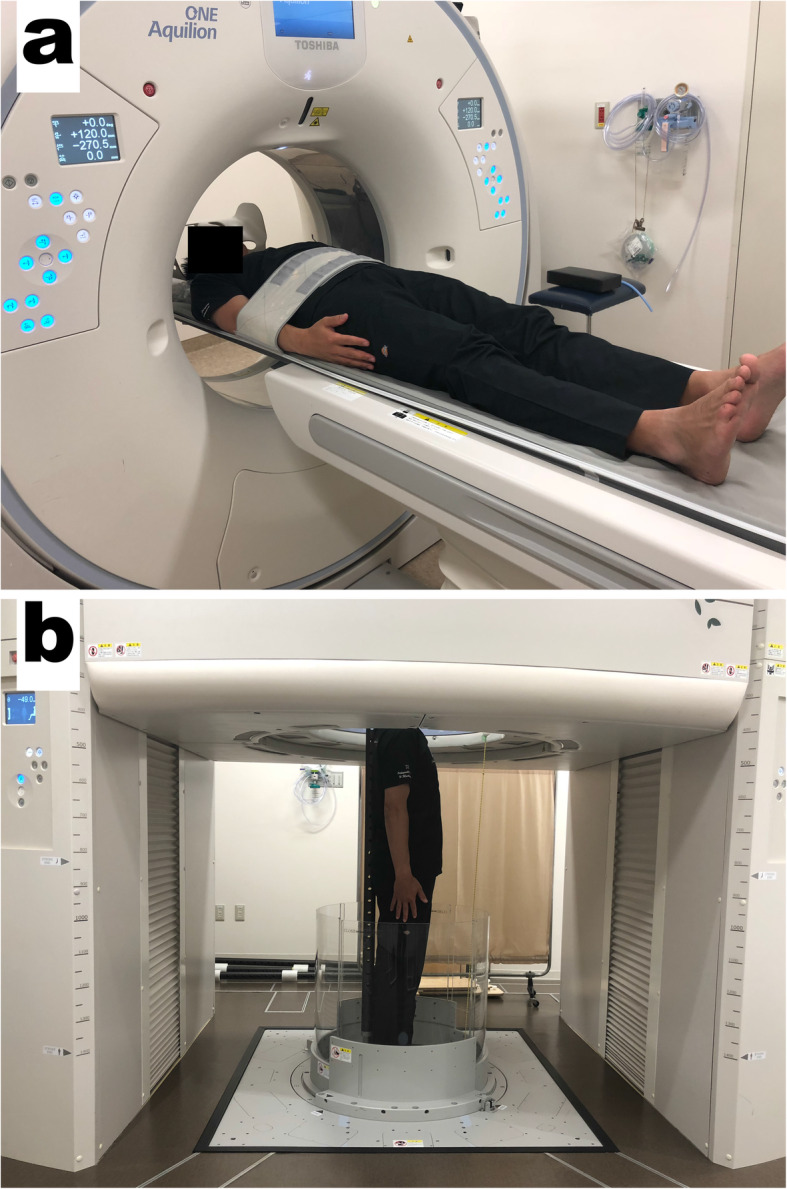
320-row conventional and upright computed tomography (CT) scanners. CT images of the bilateral shoulders were obtained with the shoulders adducted and the arms held in a neutral position, both in the supine position using a conventional scanner (**a**) and in the standing position using an upright scanner (**b**)

Imaging of the bilateral shoulders was acquired for each participant using a conventional 320-detector-row CT scanner (Aquilion ONE; Canon Medical Systems Corporation, Otawara, Japan) in the supine position and an upright CT scanner (prototype TSX-401R; Canon Medical Systems Corporation, Otawara, Japan) in the standing position on the same day (Fig. 1a, b). During acquisition, the shoulders were adducted and the arms held in neutral position. The CT data were accumulated in Digital Imaging and Communication in Medicine (DICOM) data format.

### Measurement of AHD

**Fig. 2:**
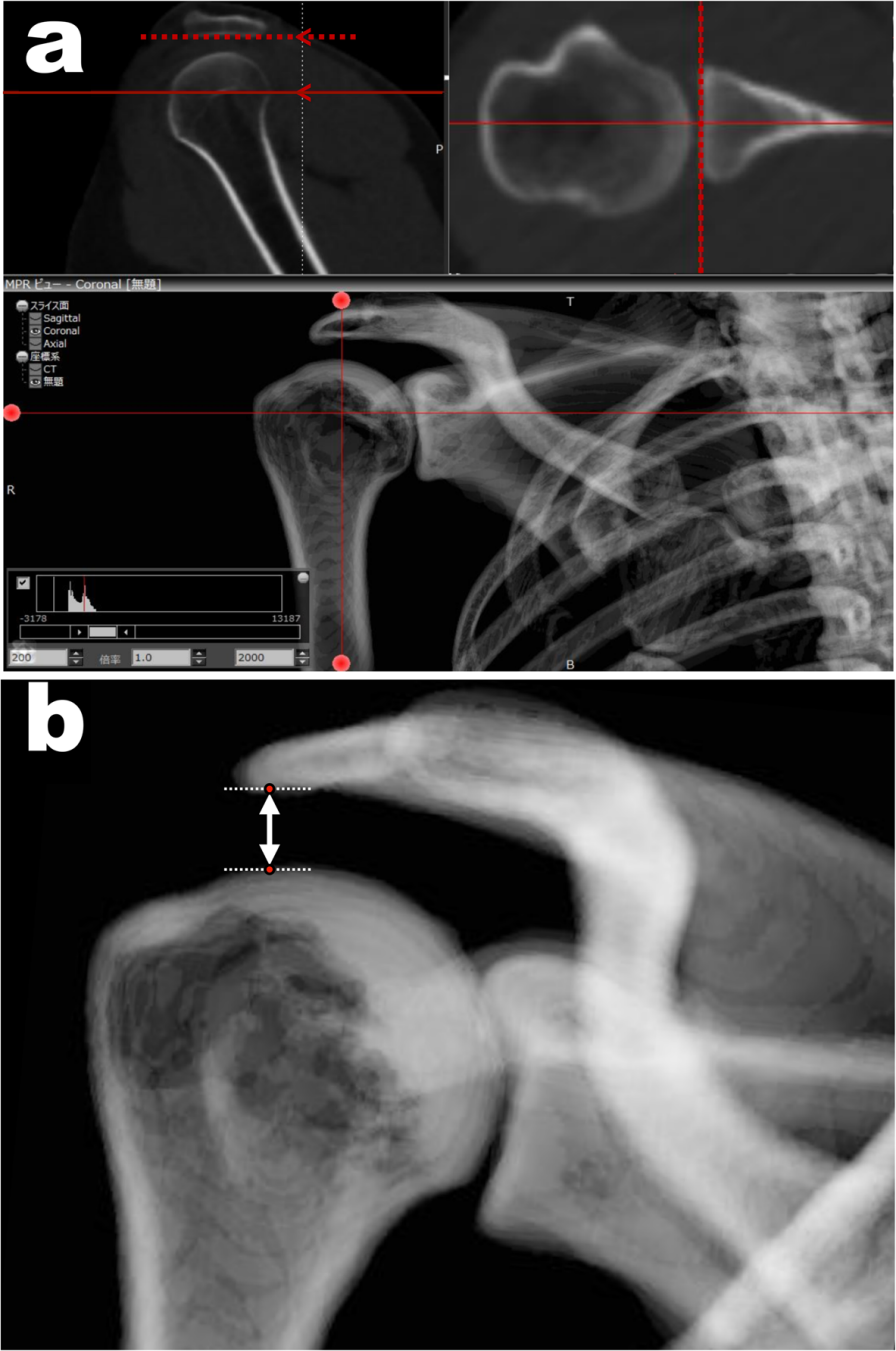
The process of measuring the two-dimensional acromiohumeral distances (2D AHD). **a** Creating true anteroposterior shoulder digitally reconstructed radiograph (DRR) image from the CT data using ZedView software. Scapula rotation was corrected to align parallel to the inferior aspect of the acromion in sagittal view and align parallel to the face of the glenoid in axial view. **b** The acromiohumeral distance (AHD) was measured using a two-dimensional (2D) approach on DRR images of the anteroposterior shoulder reconstructed from the CT scans. The 2D AHD was defined as the shortest distance from the dense cortical bone at the inferior aspect of the acromion to the most proximal articular cortex of the humeral head (white line with arrow)

AHD was measured in 2D from digitally reconstructed radiograph (DRR) images of the anteroposterior (AP) shoulder radiographs, reconstructed from the DICOM data using ZedView software (version 12.5.0; LEXI, Tokyo, Japan). Multiplanar reformatting was used to create true AP shoulder DRR images, and scapula rotation was corrected for each individual glenoid version to provide a true AP view aligned parallel to the face of the glenoid (Fig. 2a). The bone window width set as 2000 Hounsfield units (HU) and the window level as 200 HU. The AHD was measured from the dense cortical bone at the inferior aspect of the acromion to the most proximal articular cortex of the humeral head, with the shortest distance recorded as the 2D AHD (Fig. 2b) [2, 3, 5, 13].

**Fig. 3:**
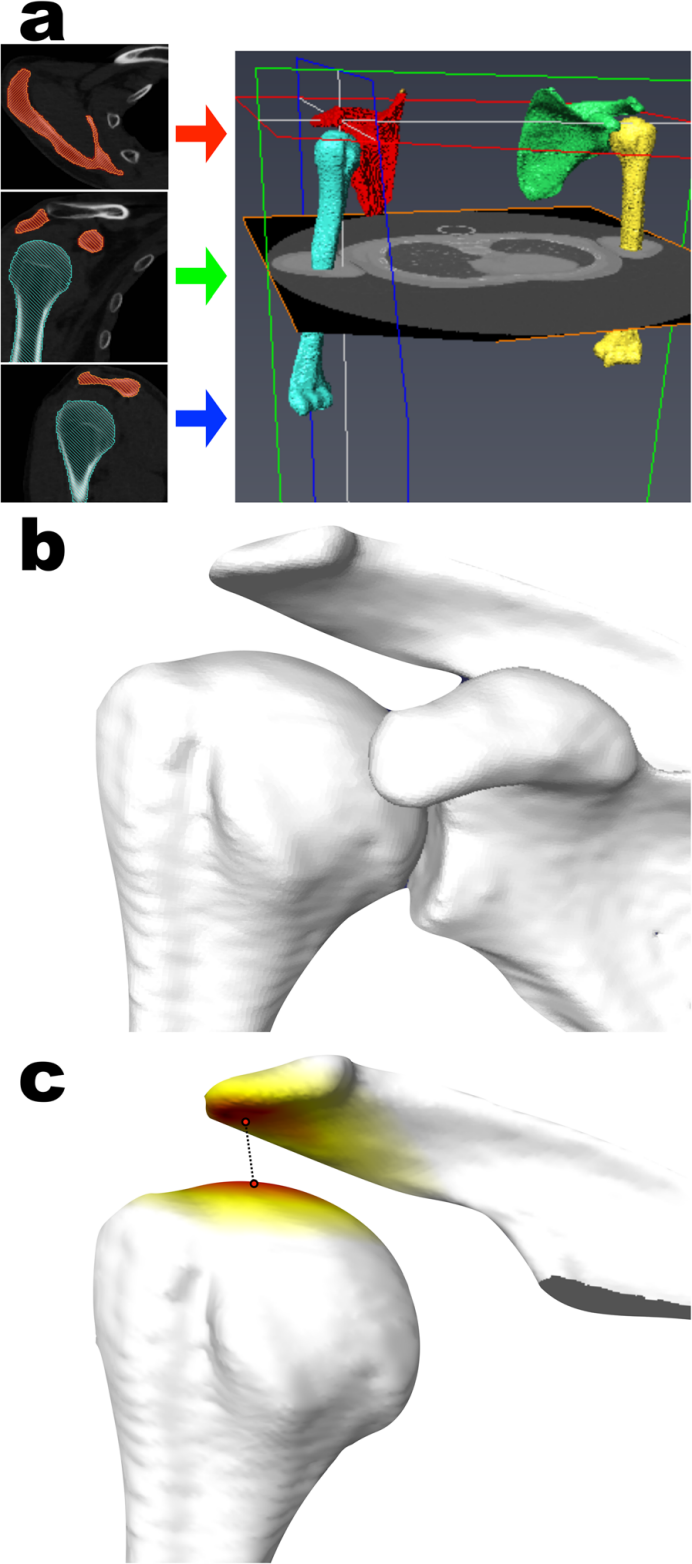
The process of calculating the three-dimensional acromiohumeral distances (3D AHD). **a** Creating 3D surface models of the scapula and humerus from the CT data to observe three views of multiplanar reformatting carefully using AVIZO software. **b** Bone surface model of the scapula and proximal humerus. **c** After removing the glenoid and coracoid parts of the surface model, the 3D AHD was automatically measured as the minimum distance between the acromion and humeral head on the software. The red areas indicate where the distance between the acromion and the humeral head is at a minimum

Furthermore, 3D bone surface models of the acromion and humeral head, which were extracted from the DICOM data using AVIZO software (version 9.3.0; Maxnet, Tokyo, Japan), were used for measuring the 3D AHD. Bone part segmentation was performed to observe each slice of multiplanar reformatting carefully, with the bone window width set as 2000 HU and the window level as 200 HU (Fig. 3a). The bone surface model was generated with the smoothing level setting of 1.75, and the scapula and the humerus surface models were exported as Standard Triangulated Language (STL) data (Fig. 3b). To evaluate the 3D distance between the acromion and the humeral head, the STL data for the glenoid and coracoid parts of the scapula surface models were removed using Meshlab software (version 1.3.3; ISTI, Pisa, Italy). The minimum distance of these two bone surface models was automatically computed as the Hausdorff distance using the Meshlab software [14–16]. This distance indicates that any point in the acromion surface model can be reached at any point in the humeral head surface model by advancing at least the distance. The closest points between the acromion and humeral head were generated using the Hausdorff sampling filter, and the value of the minimum vertex quality was recorded as the 3D AHD (Fig. 3c).

### Statistical analysis

SPSS Statistics 25.0.0.0 software (IBM Corp., Armonk, NY, USA) was used for the statistical analyses. The intra- and inter-observer reliabilities for the 2D and 3D AHD values were assessed by calculating intraclass correlation coefficient (ICC) based on 20 randomly selected shoulders. The measurements were made blind by two shoulder surgeons (ICC model 2,1) and repeated after a 3-month interval by one shoulder surgeon (ICC model 1,1). After the reliabilities were determined to be acceptable, the 2D and 3D AHD values for all 166 shoulders were assessed by a single shoulder surgeon.

The AHD data did not present normal distribution using the Shapiro–Wilk test (*P* < 0.05), and nonparametric tests were performed. The differences in age, height, weight, BMI, and AHD values between the men and women were assessed using the Mann–Whitney *U* test. The differences in the AHD values between the 2D and 3D measurements and between those measured in the supine and standing positions were evaluated using Wilcoxon signed-rank tests. Correlations in AHD values between the right and left shoulders were analyzed using Spearman’s rank correlation analysis. The relationships between the AHD values and the participants’ heights, weights, and BMI were also evaluated using Spearman’s rank correlation analysis. The significance level was set at 0.05 for all the analyses.

## Results

The intra- and inter-observer correlation coefficients for the 2D AHD measurements were 0.865 (95% confidence interval [CI], 0.695–0.944) and 0.831 (95% CI, 0.662–0.930), respectively. Those for the 3D AHD measurements were 0.979 (95% CI, 0.940–0.992) and 0.992 (95% CI, 0.981–0.997), respectively. These results confirmed that the measurements of 2D and 3D AHD were highly reproducible.

**Fig. 4:**
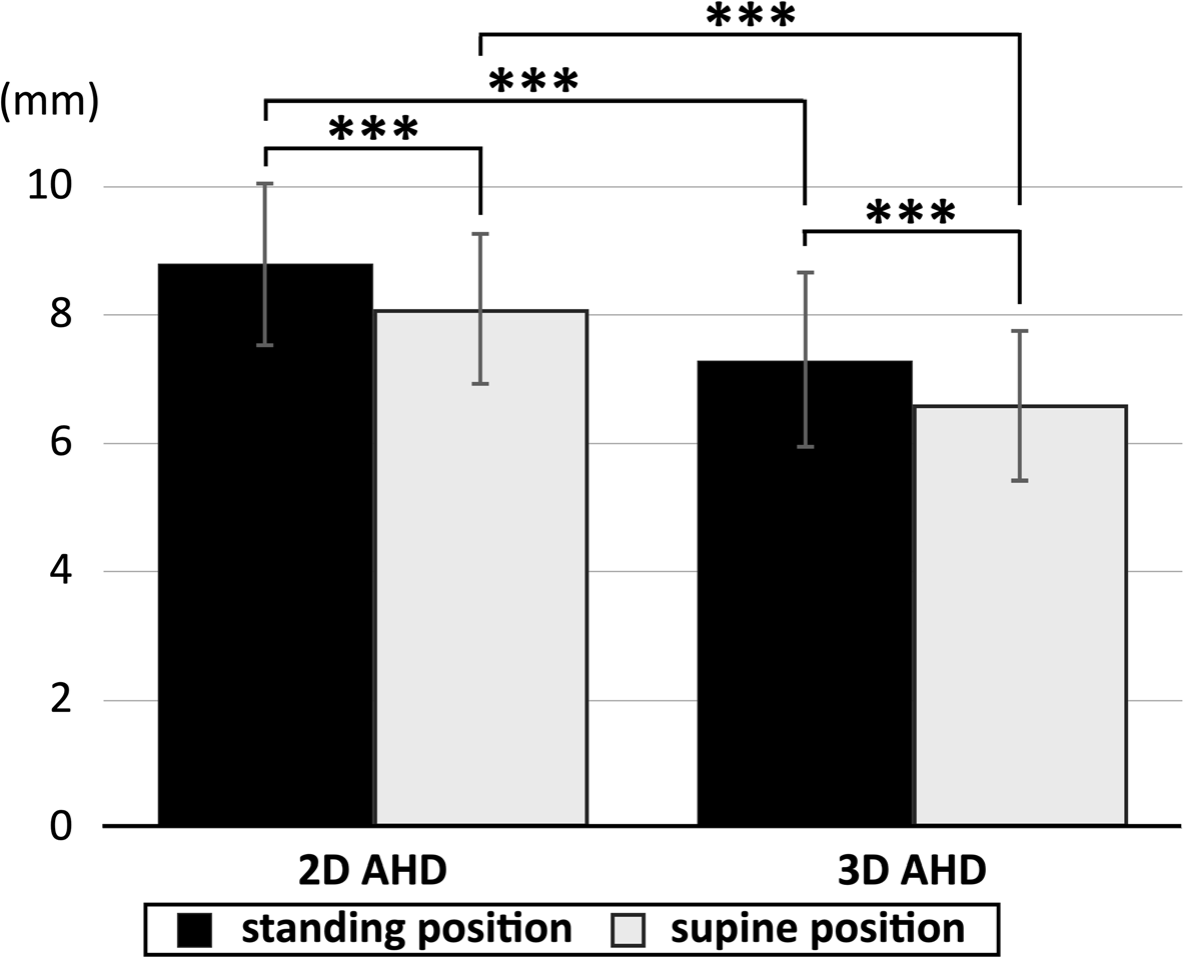
Differences in acromiohumeral distances (AHD) between 2D and 3D measurements and between the supine and standing positions. The 3D AHD values were significantly lower than the 2D AHD values in both the standing and supine positions. The 2D and 3D AHD values were significantly lower in the supine position than in the standing position. ***P < 0.001

**Fig. 5:**
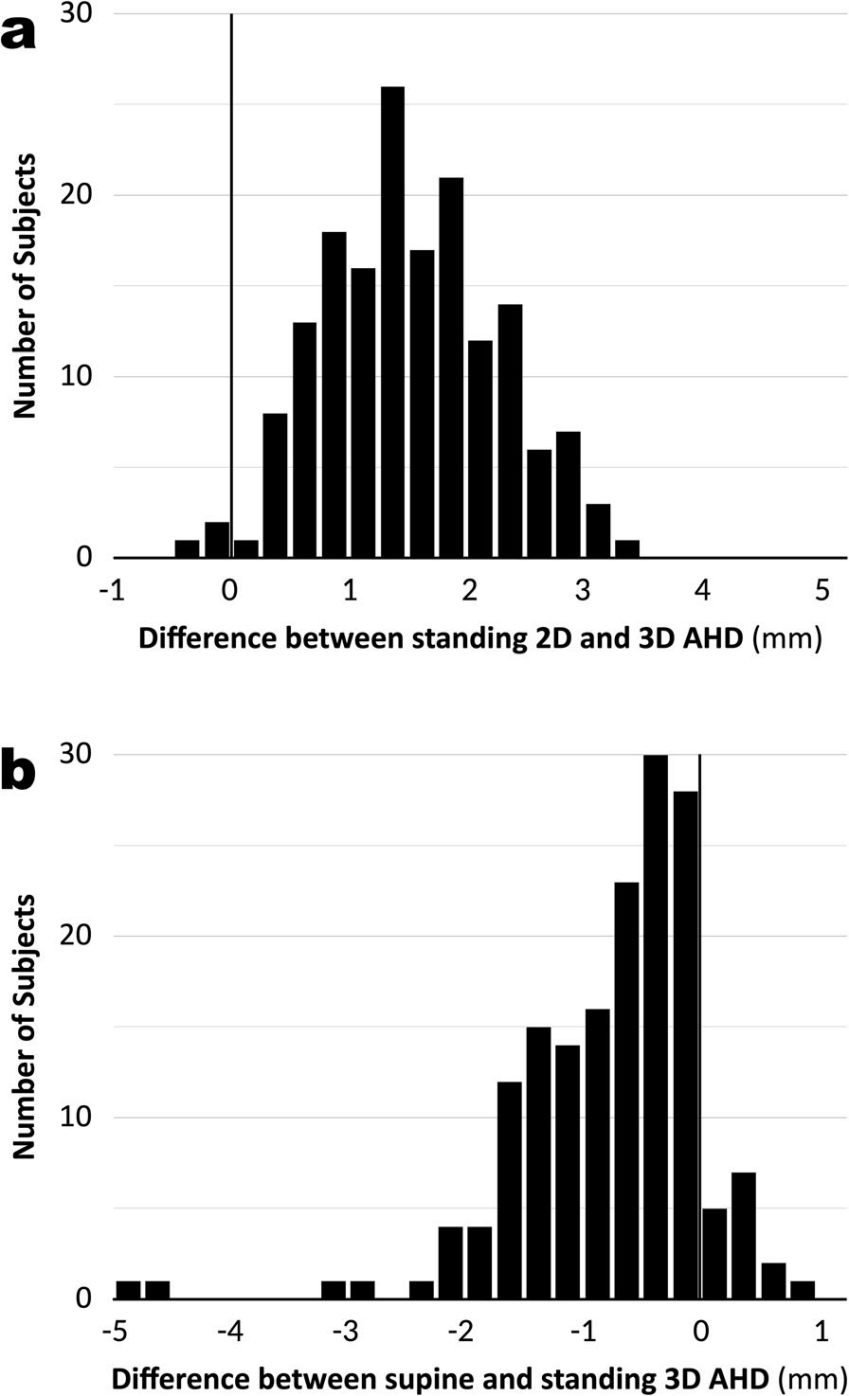
Histogram of the individual differences in acromiohumeral distance (AHD). **a** Histogram of the individual differences in AHD in the standing position between the 2D and 3D measurements. Positive values indicate the 2D value is greater than the 3D value. **b** Histogram of the individual differences in 3D AHD between the supine and standing positions. Positive values indicate that the standing value is greater than the supine value. The differences varied widely

The mean values for the 2D AHDs were significantly higher in the standing position than in the supine position, at 8.8 ± 1.3 mm (range, 5.9–15.4 mm) and 8.1 ± 1.2 mm (range, 5.3–14.3 mm), respectively; similarly, the mean values for the 3D AHDs were significantly higher in the standing position than in the supine position, at 7.3 ± 1.4 mm (range, 4.7–14.0 mm) and 6.6 ± 1.2 mm (range, 4.4–13.7 mm), respectively (Fig. 4). The 3D AHD values were significantly lower than those for the 2D AHDs in both the standing and supine positions (both *P* < 0.001). The individual differences between the 2D and 3D AHD values obtained in the standing position ranged widely from – 0.4 to 3.3 mm (Fig. 5a). The individual differences between the 3D AHD values obtained in the supine position and those obtained in the standing position showed even more variation, from – 4.9 to 0.8 mm (Fig. 5b).

The mean values of both the 2D and the 3D AHD measurements were significantly lower for the women than for the men in both the standing and supine positions (Table 1).

**Table 1:** Participant characteristics and two-dimensional (2D) and three-dimensional (3D) measurements of the acromiohumeral distance (AHD) made from images acquired in the standing and supine positions

		All, *N* = 83	Male, *n* = 31	Female, *n* = 52	*P* (sex difference)
Characteristic	Age, years	40.2 ± 5.8	39.4 ± 4.9	40.7 ± 6.2	.154
	Height, cm	163.0 ± 8.8	171.5 ± 6.0	157.9 ± 5.6	< .001***
	Weight, kg	59.7 ± 12.5	69.7 ± 11.8	53.8 ± 8.5	< .001***
	BMI, kg/m^2^	22.4 ± 3.7	23.7 ± 3.8	21.6 ± 3.5	< .001***
2D AHD, mm	Standing position	8.8 ± 1.3	9.1 ± 1.4	8.7 ± 1.3	.034*
	Supine position	8.1 ± 1.2	8.5 ± 1.3	7.8 ± 1.1	.001**
3D AHD, mm	Standing position	7.3 ± 1.4	7.7 ± 1.5	7.1 ± 1.4	.003**
	Supine position	6.6 ± 1.2	7.0 ± 1.4	6.3 ± 1.1	< .001 ***

The data are presented as mean ± standard deviation. *BMI* body mass index, *AHD* acromiohumeral distance. *P < .05, **P < .01, ***P < .001

**Fig. 6:**
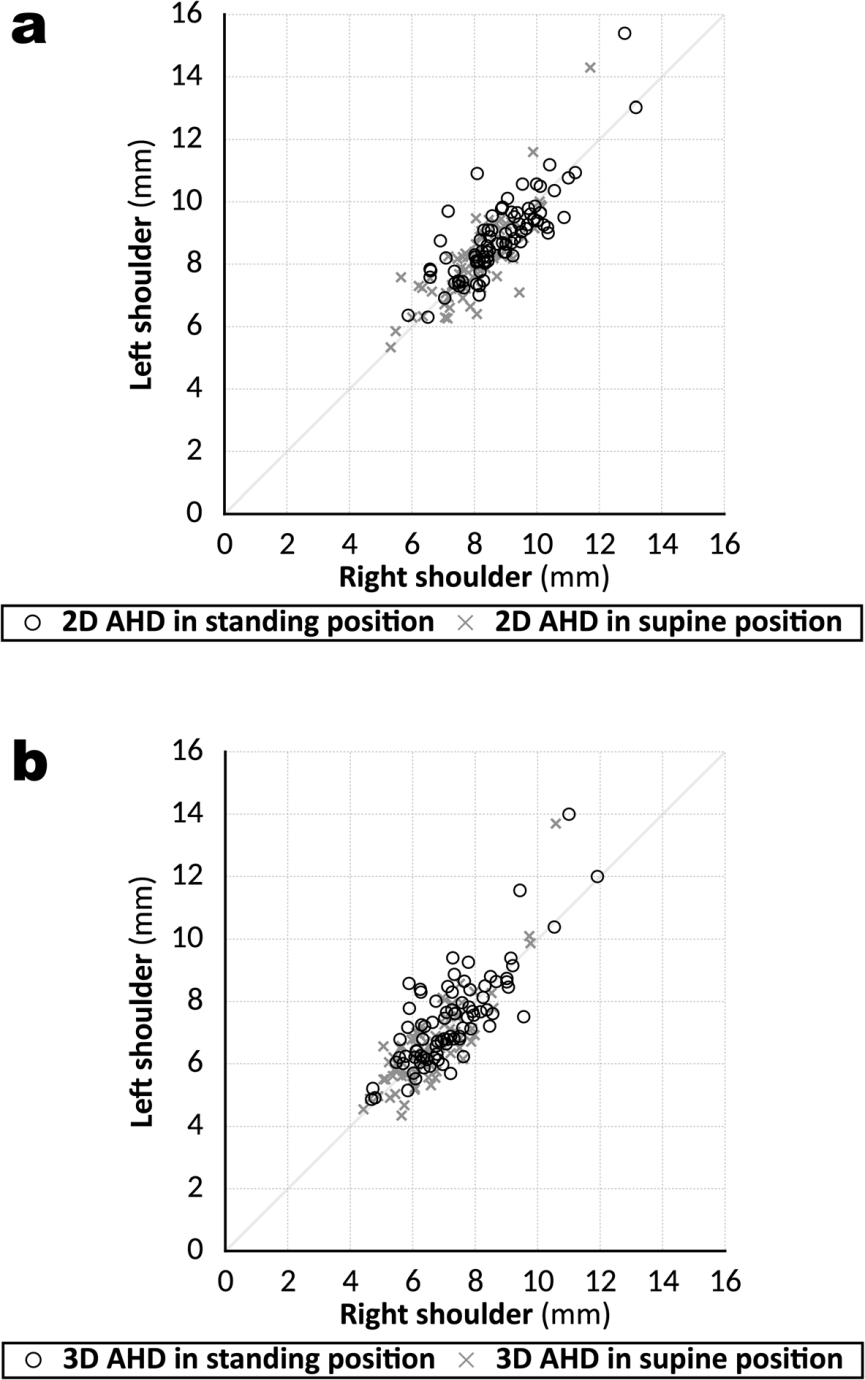
Linear regression plots of acromiohumeral distance (AHD) values compared between the right and left shoulders. **a** Linear regression plots of acromiohumeral distance (AHD) values measured in two dimensions in the supine and standing positions, compared between the right and left shoulders. The values showed a strong correlation between the sides (standing: *R* = 0.794, *P* < 0.001; supine: *R* = 0.780, *P* < 0.001). **b** Linear regression plots for AHD values measured in three dimensions in the supine and standing positions, compared between the right and left shoulders. The values showed a strong correlation between the sides (standing: *R* = 0.711, *P* < 0.001; supine: *R* = 0.742, *P* < 0.001)

Strong correlations were observed between the right and left shoulders for both the 2D and 3D AHDs, standing and supine (2D AHD: standing, *R* = 0.794, *P* < 0.001; supine, *R* = 0.780, *P* < 0.001; 3D AHD: standing, *R* = 0.711, *P* < 0.001; supine, *R* = 0.742, *P* < 0.001) (Fig. 6a,b). There was a weak correlation between the participants’ height and the standing 3D AHD values, but not with any of the other AHD values (2D AHD: standing, *R* = 0.098, *P* = 0.211; supine, *R* = 0.137, *P* = 0.079; 3D AHD: standing, *R* = 0.161, *P* = 0.038; supine, *R* = 0.149, *P* = 0.055). Weak correlations were observed between the ADH values and the participants’ weight (2D AHD: standing, *R* = 0.299, *P* < 0.001; supine, *R* = 0.386, *P* < 0.001; 3D AHD: standing, *R* = 0.319, *P* < 0.001; supine, *R* = 0.310, *P* < 0.001) and BMI (2D AHD: standing, *R* = 0.303, *P* < 0.001; supine, *R* = 0.386, *P* < 0.001; 3D AHD: standing, *R* = 0.284, *P* < 0.001; supine, *R* = 0.290, *P* < 0.001).

## Discussion

This study evaluated the shortest distance between the acromion and humeral head in the standing position by using a newly developed upright CT scanner with a 3D approach [8, 9]. Measuring 3D AHD while standing straight was feasible, and the mean value of the 3D AHD of healthy participants without previous injuries was 7.3 mm, ranging from 4.7 to 14.0 mm. The AHD was greater in men than in women. These results were compared with values of AHD evaluated using a 2D method in both the standing and supine positions and using a 3D method in the supine position. The 2D measurements were significantly higher than the 3D measurements, and the supine measurements were significantly lower than the standing measurements. Hence, the 2D analysis can overestimate the shortest distance between the acromion and the humeral head, and assessment in the supine position can underestimate the AHD compared with that in the standing position.

The normal value of AHD calculated using anteroposterior radiographs has been reported as 6–14 mm [1–4, 17, 18]. The values of 2D AHD evaluated in the present study were consistent with these previous reports, but they were significantly greater than the values for 3D AHD, which are automatically computed assessment of the minimum distance between the 3D surfaces of the acromion and the humeral head. The individual differences between the 2D and 3D AHD values measured in the standing position varied widely by up to 3.3 mm. According to the present findings, conventional 2D analysis cannot detect the actual shortest points of the acromion and humeral head because the inferior surfaces of these have a curved structure, which can be observed using 3D imaging [5].

The values of both 2D and 3D AHDs in the standing position were significantly higher than those in the supine position. In the standing position, gravity is likely to result in the humeral head moving downwards. The individual differences in 3D AHD between the standing and supine positions also varied widely, by up to 4.9 mm, suggesting that the alignment changes in the glenohumeral joint between positions vary between individuals. In cases of rotator cuff tear, the patients often suffer from pain at night and sleep disturbance when lying in the supine position [19]; therefore, Railhac et al. [20] stated that measuring AHD in the supine position is useful in detecting a rotator cuff tear. Different positions can change the subacromial pressure [21, 22], and the narrowing of the AHD in the supine position might increase pain.

This was the first study to evaluate the AHD in the standing position using CT imaging. Saupe et al. [13] and Werner et al. [23] reported that 3D AHD measurements on magnetic resonance imaging acquired in the supine position were 2.8 mm and 0.6 mm lower, respectively, than 2D AHD measurements made on radiographs in the standing position. Similarly, Ongbumrungphan et al. [7] reported that 3D AHD measurements on CT imaging acquired in the supine position were 1.7 mm lower than 2D AHD radiograph measurements in the standing position. However, the insight into whether differences in human position or those between 2D and 3D analyses caused AHD variability still remains unclear. This study indicated that these differences may be caused by a combination of AHD overestimation by 2D analysis and AHD underestimation in the supine position. We believe that 3D AHD measured in the standing position, which reflects the actual distance between the acromion and the humeral head during daily activity, would be beneficial and helpful for clarifying the complex function of the shoulder in future studies.

Similar to the past reports [3, 24], the values of AHD in the present study varied greatly between individuals, and the AHD values were smaller in women than in men. The values of 3D AHD had a weak but significant correlation with the participants’ height, weight, and BMI. This showed that the values of AHD differ with individual physical status, but that other factors including the shape of acromion and rotation of the scapula affect the values. The values of AHD were strongly correlated between sides, confirming that the AHD of the contralateral shoulder can act as a reference when assessing the AHD in cases of unilateral shoulder pathology.

The present study had several limitations. First, although the participants were healthy volunteers without any shoulder symptoms, we could not evaluate whether they had asymptomatic rotator cuff tears. The age of the volunteers was limited to 30–49 years because of high correlation between the onset of rotator cuff tears and increasing age and because rotator cuff tears are clinically associated with lower AHD values. Yamaguchi et al. [12] reported that the mean age for individuals with no rotator cuff tear was 48.7 years, whereas for those with a unilateral tear it was 58.7 years, and for those with a bilateral tear it was 67.8 years. We excluded the volunteers over 50 years of age to ensure we avoided including shoulders with asymptomatic rotator cuff tear or other shoulder pathology. The comparisons between 2D and 3D measurements and between positions in the present study may differ from those that would be obtained for shoulders with rotator cuff tear; further studies are needed to investigate this. The measurement of 2D AHD might be another limitation. This was evaluated on DRR images reconstructed from CT data. DRR can be used to obtain a true anteroposterior view [25], and a past validation study [26] demonstrated that DRR can substitute for radiographs; however, the images obtained in this way may differ from the conventional radiographs used in previous 2D analyses.

## Conclusions

This study evaluated the 3D AHD of normal shoulders in the standing position by using an upright CT scanner. The present results indicated that, compared with the measurement of AHD made in the standing position with 3D analysis, measurements with 2D analysis can overestimate the value of AHD and assessments in the supine position can underestimate the value.

## Data Availability

The datasets used and/or analyzed during the current study are available from the corresponding author on reasonable request.

## Abbreviations

AHD: Acromiohumeral distance
BMI: Body mass index
CT: Computed tomography
DICOM: Digital Imaging and Communication in Medicine
DRR: Digitally reconstructed radiograph
HU: Hounsfield units
ICC: Intraclass correlation coefficient
STL: Standard Triangulated Language
2D: Two-dimensional
3D: Three-dimensional
